# The Use of Technology in the Prevention of Infections Associated with Urinary Catheterization

**DOI:** 10.3390/nursrep14040284

**Published:** 2024-12-07

**Authors:** Bruna Raquel Fonseca, Maura Filipa Silva, Rogério Ferrinho Ferreira, Sofia Cabecinhas de Sá, Teresa Dionísio Mestre, Marta Sofia Catarino

**Affiliations:** 1 School of Health Sciences, Polytechnic Institute of Beja, 7800-111 Beja, Portugal; 61411@chln.min-saude.pt (B.R.F.); ferrinho.ferreira@ipbeja.pt (R.F.F.); sofia.sa@ipbeja.pt (S.C.d.S.); teresa.dionisio@ipbeja.pt (T.D.M.); 2 Santa Maria Local Health Unit, Public Business Entity, 1649-035 Lisboa, Portugal; 3 Barreiro-Montijo Hospital Center, Public Business Entity, 2830-092 Barreiro, Portugal; 4 Health Department, Polytechnic Institute of Beja, 7800-111 Beja, Portugal; 5 Educational Sciences, Health Department Polytechnic Institute of Beja, 7800-111 Beja, Portugal; 6 Baixo Alentejo Local Health Unit, Public Business Entity, 7800-309 Beja, Portugal; 7 Comprehensive Health Research Centre (CHRC), Universidade Nova de Lisboa, 1099-085 Lisboa, Portugal; 8 Center of Interdisciplinary Research in Health (CIIS), Institute of Health Sciences (ICS), Universidade Católica Portuguesa, 1649-023 Lisboa, Portugal; 9 Faculty of Health Sciences and Nursing (FHSN), Universidade Católica Portuguesa, 1649-023 Lisboa, Portugal

**Keywords:** catheter-associated urinary tract infections, catheter indwelling, electronic devices, nursing, technology

## Abstract

(1) Background: Urinary tract infections (UTIs) are caused by the proliferation of pathogenic microorganisms, and they are the second most common hospital-acquired infections, with catheter-associated UTIs (CAUTIs) accounting for about 40% of these nosocomial infections. This review aims to identify the impact of technology on preventing infections in patients with urinary catheters; (2) Methods: The search was conducted in April 2024 through the EBSCOhost platform, with access to the American Search Complete, CINHAL Ultimate, Medline databases, and through the Scopus database; (3) Results: In total were included eight articles in this review. Technology interventions can significantly reduce the incidence of CAUTIs, decrease the duration of catheter use, improve diagnosis, and enhance patient safety; (4) Conclusions: Technological advancements show significant benefits in reducing infection rates and improving patient outcomes, like shorter hospital stays and comfort. Multidisciplinary approaches and educational strategies are essential to maximize these benefits.

## 1. Introduction

Urinary dysfunctions significantly affect the quality of life of millions of people, ranging from school-age children to the elderly, who may experience urinary incontinence. Some urinary system diseases require monitoring of bladder volume, necessitating the use of easy-to-handle and accurate measurement devices [1].

To evaluate bladder status and urine volume, both invasive and non-invasive methods can be employed. Catheterization, an invasive technique involving the insertion of a catheter through the urethra into the bladder, allows for precise measurement of urine volume. However, it is a painful procedure that limits patient mobility in daily life and can lead to infections or damage to the urinary tract [1].

It is estimated that 5–10% of patients acquire one or more nosocomial infections during hospitalization, with catheter-associated urinary tract infections (CAUTIs) being among the most common. CAUTIs account for approximately 40% of all hospital infections and contribute to prolonged hospital stays [2].

In recent years, the use of urinary catheters has increased significantly, often due to inappropriate or unnecessary indications in patients who do not meet the criteria for catheterization [2]. Long-term catheter use is an independent risk factor for the development of multidrug-resistant infections and serves as a source of cross-contamination for other hospitalized patients [2,3]. This highlights the importance of continuously reassessing catheter maintenance [3].

Approximately 50% of patients with long-term catheters experience obstruction within the first six months, which can lead to severe complications, such as pyelonephritis and sepsis. These complications are often caused by biofilms, sediment in the catheter tubing, or catheter compression [4].

Technological innovations; for example, electronic alert systems for catheter removal or non-invasive bladder monitoring devices, have significantly improved and management of urinary catheters by ensuring appropriate catheter duration, enhancing patient comfort, and reducing the risk of infections. Therefore, the following research question was defined: “What is the impact of technology on infection prevention in patients undergoing urinary catheterization?”.

## 2. Materials and Methods

Methodologically, this review was structured considering the following five stages: (1) Determination of the inclusion and exclusion criteria for the studies; (2) Definition of the descriptors to be extracted for data organization; (3) Analysis of the previous studies selected for the review; (4) Discussion of the results obtained; (5) Presentation of the review conclusions.

### 2.1. Research Question

The definition of the research question was elaborated through the PICOD methodological application: “P” (Population)––adult patients with urinary catheterization; “I” (Intervention)––use of technology in healthcare; “C” (Comparison)––not applicable; “O” (Outcome)––infection prevention; “D” (Design”)––Integrative Literature Review (ILR).

### 2.2. Inclusion and Exclusion Criteria

The inclusion criteria for this integrative literature review (ILR) were as follows: primary studies using qualitative, quantitative, or mixed methods including randomized and non-randomized trials, as well as systematic and integrative literature reviews. Only studies published between 2018 and 2024 were considered.

The selected articles had to be available in full text in Portuguese, English, and Spanish. The review specifically focused on examining the impact of technology on infection prevention in patients with urinary catheters. Therefore, studies were required to involve adult patients undergoing urinary catheterization to ensure their relevance to the review’s objective.

Exclusion criteria included articles that did not align with the theme of technologies’ impact on infection prevention in urinary catheter patients. Studies with unclear or unspecified methodologies, duplicates, or those published before 2018 were also excluded. Also, articles that did not pertain to hospital or homecare settings were excluded, ensuring that only studies with relevant contexts and populations were considered. This approach helped maintain the focus and relevance of the review.

### 2.3. Search Strategy

The bibliographic search for articles was conducted in April 2024 using the databases Academic Search Complete by EBSCOhost Web and CINAHL Ultimate by ESBCOhost Web, Medline by EBSCOhost Web, Medline by PubMed, and Scopus. To access these databases, keywords, truncations, Medical Subject Headings, and Boolean operators “AND” and “OR” were employed. The following table (Table 1) presents a search equation used for the Medline database via PubMed.

A total of 192 articles were retrieved from the following databases: 23 articles from Academic Search Complete; 10 from CINHAL Ultimate; 29 from Medline Ultimate; 37 from Medline via PubMed; 93 articles from Scopus. These articles were exported to the EndNote Web 21 platform [5], where the duplicates removal function was used, resulting in the elimination of 50 duplicate articles.

Title and abstract screening was then performed, leading to the exclusion of 111 articles. A total of 31 were then assessed for eligibility by reading the full texts.

At this stage, 23 articles were excluded for not meeting the study’s criteria. In the end, a total of eight articles were included in this review, consisting of seven primary studies and one ILR.

**Figure 1:**
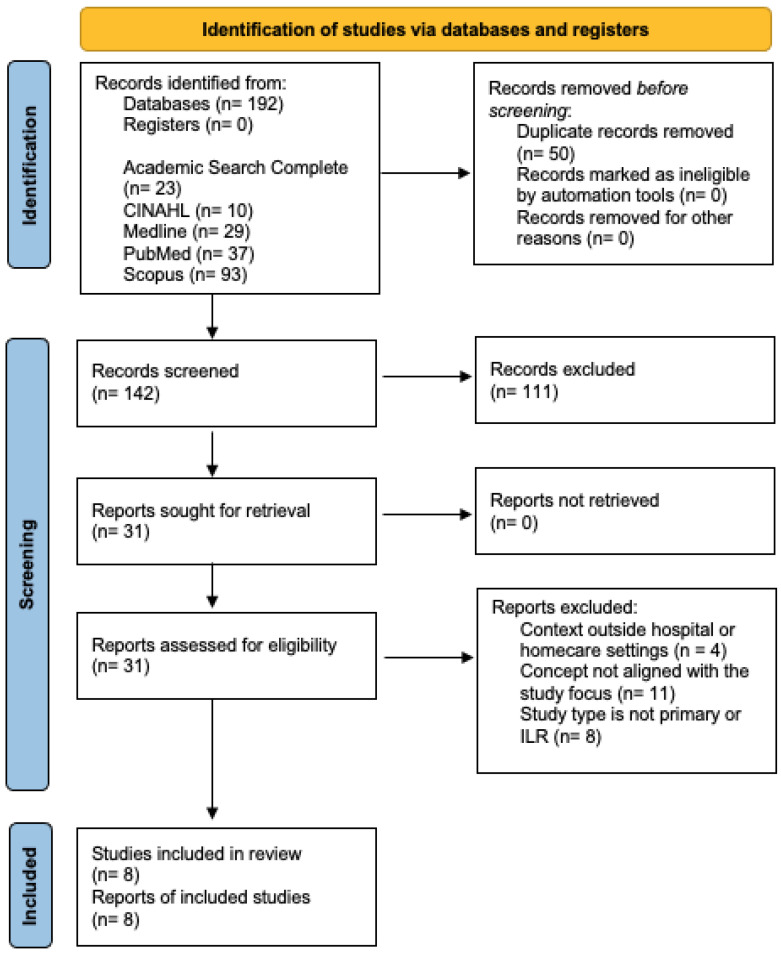
PRISMA of the study selection procedure.

This selection process is illustrated in the PRISMA flow diagram on Figure 1 (Preferred Reporting Items for Systematic Reviews and Meta-Analyses), as shown in Figure 1.

To ensure the credibility, quality, and level of evidence of the selected primary studies, the Joanna Briggs Institute (JBI) method [6] was used. This provides a systematic framework for critically assessing studies, contributing to rigorous evidence-based scientific research practices.

Since this review was an ILR, the JBI method was the only approach used to evaluate the level of evidence. No other quality assessments of the articles were applied to the included studies.

Table 2 presents the level of evidence of the articles used in this ILR, according to the JBI method.

## 3. Results

In this ILR, eight studies published between 2018 and 2024 were included, focusing on the impact of technologies in preventing infections associated with urinary catheterization in hospitalized patients. These studies were selected based on the inclusion criteria, including thematic relevance, study designs, and the clinical applicability of the evaluated technologies [2,3,4,7,8,9,10].

### 3.1. Study Descriptions

The selected studies varied in terms of design and objectives. There were three observational studies (two prospective and one retrospective), along with two experimental studies (one quasi-experimental and one randomized controlled trial), one diagnostic evaluation study, one mixed-methods study, and one integrative literature review.

These studies were conducted in diverse hospital settings across different countries, including the United States, Australia, Japan, and Switzerland. The sample sizes ranged from small cohorts of 26 nurses [4] to large groups of 1167 patients [9].

### 3.2. Main Findings

The selected studies demonstrated a significant impact of various technologies on reducing infections associated with urinary catheterization in hospitalized patients; for instance, electronic systems, non-invasive monitoring, biomimetic technology, and innovative diagnostic tools.

Topal et al. [2] found that a computerized feedback system for physicians and a nurse-directed protocol reduced catheter use and led to a notable decrease in CAUTIs. The adoption of this technology was well received by the nursing staff when compared with the traditional paper-based methods.

In addition to feedback systems, other technologies have shown promising results in infection prevention. Dong et al. [7] investigated a diagnostic tool using flow microimaging and artificial intelligence for rapid UTI diagnosis. This study concluded that this technology demonstrated a high specificity (96.5%) and reasonable sensitivity (72.9%), suggesting its potential to reduce unnecessary urine cultures and improve diagnostic efficiency.

Similarly, Mitchell et al. [9] and Fasugba et al. [3] assessed the efficacy of an electronic reminder system (CATH TAG) in reducing catheterization duration. Their studies demonstrated a significant reduction in the duration of catheter use, which corresponded with a 23% decrease in CAUTI incidence, reinforcing the utility of these reminders in clinical practice.

On the other hand, Xu et al. [10] explored the use of biometric urine flow control technology focusing on preserving bladder function in patients with long-term catheterization. This innovative approach aims to improve patient outcomes and potentially reduce infection risk associated with prolonged catheter use.

To further demonstrate these findings, the data collected on the characteristics of the studies are synthesized in Table 3, which provides a clearer overview and facilitates the understanding of these positive outcomes.

### 3.3. Interpretation and Conclusions

Several technologies have shown promise in reducing CAUTIs, either directly or indirectly, while also providing gains in other areas of patient care. Topal et al. [2] demonstrated that a computerized feedback system, combined with nurse-directed protocols, resulted in a significant reduction in both catheter use and CAUTI incidence. By providing real-time feedback to physicians and nurses, this system promoted, in a more timely manner, the removal of catheters, which minimized the risk of infections. This success demonstrates how integrating technological solutions with evidence-based practices can substantially improve clinical outcomes.

The studies developed by Mitchell et al. [9] and Fasugba et al. [3] found that the electronic reminder system, CATH TAG, significantly reduced the duration of catheterization and the subsequent incidence of CAUTIs. This system can effectively remind healthcare professionals to remove the catheter at appropriate intervals. The impact of this technology in reducing CAUTI rates emphasizes the importance of continuous monitoring and reminders in clinical practice to ensure patient safety.

In contrast, non-invasive urine volume monitoring technologies have proven effective in managing patients in less critical settings. Although they do not directly prevent CAUTIs, these systems reduce the need for frequent catheter adjustments, which can decrease the risk of complications (e.g., UTIs).

Technologies focused on preserving bladder function, such as urethral sphincter simulation, help to improve patient outcomes by reducing discomfort and maintaining urinary function in long-term catheter users. In this case, these advancements may not directly target CAUTIs, but they can contribute to improving the overall quality of care and mitigating other complications related to prolonged catheterization.

All of these technological advancements underscore the importance of integrating innovative solutions into clinical practice, whether focusing on preventing CAUTIs or increasing other aspects of healthcare services.

Table 4 presents a detailed summary of these findings, assessing the impact of each technology on CAUTI prevention as reported in each study. It indicates whether each article supports the effectiveness of these technologies in reducing CAUTI incidence, thereby offering a comprehensive overview of their preventive impact across various healthcare settings and study designs.

## 4. Discussion

The findings of this review offer valuable insights into the use of long-term urinary catheters in both hospital and homecare settings, emphasizing complications like CAUTIs and highlighting the benefits of technologies, such as real-time monitoring systems and antimicrobial catheters in mitigating these risks. The results align with previous studies, reinforcing the hypothesis that technological interventions and nurse-directed protocols can substantially improve patient outcomes by reducing CAUTIs, shortening the duration of catheter use, and enhancing overall patient safety.

In line with these findings, the studies by Mitchel et al. [9] and Fasugba et al. [3] further highlight the effectiveness of specific technological interventions; for instance, the use of the CATH TAG electronic reminder system, in reducing catheterization duration and CAUTI evidence. The reduction of 23% in catheter use duration among hospitalized patients suggests that such electronic reminder systems can significantly impact nursing practices and patient’s outcomes. This emphasizes the importance of timely catheter removal in preventing infections [3,9], and the practicality and ease of use of the CATH TAG system, coupled with its ability to alert nurses to reassess catheter necessity, demonstrates its potential as a valuable tool in clinical settings.

Similarly, Fukuda et al. [4] explored non-invasive methods for preventing or reversing blockages in long-term urinary catheters, underscoring the critical role of continuous nursing education and interprofessional collaboration in improving catheter management in home care settings. This approach aligns with the broader context of enhancing nursing practices through technology, supporting the hypothesis that well-designed applications can help nurses and caregivers, leading to better patient outcomes.

In addition, Xu et al. [10] investigated biomimetic urinary flow control (BUFC) technology which simulates urethral sphincter function to maintain bladder stability in patients with long-term catheterization. This ability of the BUFC to provide controlled urine drainage aligns with the prior studies that advocate for technologies that mimic the natural bodily functions to improve patient care outcome, further emphasizing the potential of such innovations in enhancing patient safety [10].

On the other hand, Nasrabadi et al. [1] focused on non-invasive methods for bladder volume monitoring; for example, ultrasound and infrared technology, as viable alternatives to more invasive procedures. While these methods may be less precise, their ease of use and reduced discomfort for patients make them suitable for less critical situations. These perspectives broaden the discussion on catheter management, suggesting that a range of tools, from highly advanced to simple non-invasive devices, can be integrated into patient care depending on the clinical context.

The study by Topal et al. [2] demonstrated that combining electronic alerts, nurse-directed protocols, and portable ultrasound devices led to a significant reduction in CAUTI evidence. The integration of these strategies resulted in a 51% reduction in the number of patients admitted with urinary catheterization, along with a 42% decrease during hospitalization, providing strong evidence for the effectiveness of these combined interventions.

Moreover, the 81% reduction in CAUTIs per 1000 catheter days underscores the importance of multidisciplinary approaches in improving patient safety and reducing healthcare costs. This aligns with the hypothesis that combining technological innovations with protocol-driven nursing practices can lead to improvements in clinical outcomes [2].

Additionally, the urine flow microimaging system for UTI diagnosis developed by Dong et al. [7] illustrates the potential of advanced diagnostic tools in reducing unnecessary tests and optimizing antibiotic therapy. The high specificity and sensitivity of this device offer a reliable method for early UTI detection, which is critical in preventing the progress of infections and reducing hospital resource utilization.

Finally, the importance of evidence-based nursing interventions, as highlighted by Rea et al. [8], cannot be overstated. The cloud-based software that facilitates continuous care improvement and promotes adherence to best practices exemplify the role of digital tools in modern healthcare. The agreement between Rea et al. [8] and Topal et al. [2] on the effectiveness of multidisciplinary approaches and electronic devices reinforces the need for integrated strategies to combat CAUTIs and improve patient outcomes.

In the following table (Table 5), a summary chart was created to facilitate the understanding of the written text, indicating each method addressed in each article, along with its advantages and disadvantages.

## 5. Limitations

While the benefits are clear, several challenges remain for integration into healthcare settings. Barriers to implementation include additional costs, ongoing training needs, and resistance from healthcare professionals. Many studies featured small sample sizes, limiting the generalizability of their findings. There is a need to expand sample sizes and include diverse contexts to better demonstrate the impact of technologies and address limitations.

Another significant limitation in some studies is the lack of emphasis on educating patients or caregivers about the proper care and use of these devices. Many adult patients, who are often fully conscious and autonomous in their daily activities, require proper training to ensure effective use of the technology, which can impact their outcomes. Patient perceptions of the advantages and disadvantages of these devices should also be considered during implementation, allowing for a more comprehensive assessment of their impact on patients’ quality of life.

Focusing on a specific patient group, such as the elderly, was a common approach in the studies reviewed, possibly due to the reduced activity levels in this demographic. Furthermore, limitations regarding language and publication sources, as well as the geographic distribution of studies, may influence the applicability of results across different cultural contexts and healthcare systems. The transition from theoretical knowledge to effective interventions requires careful consideration of these contextual factors, adaptation, and the development of concrete strategies to ensure successful implementation.

## 6. Conclusions

The present review conducted in this study highlighted the impact of new technological resources on improving patients’ quality of life during urinary catheterization and enhancing care practices while reducing associated infection rates.

Both early and late removal of urinary catheters can have adverse effects on patients, including the need for reinsertion, increased infection risk, and decreased quality of life. Studies have confirmed that UTIs are among the most frequent complications in hospital settings, linked to urinary catheters. Symptomatic infections can cause fever, pyuria, and even more severe outcomes like sepsis and death.

The studies included in this ILR generally demonstrated that the use of these devices contributes to reducing the burden on healthcare systems, particularly in terms of financial resources, shorter hospital stays, and enhanced patient comfort.

Despite these technological innovations, challenges remain in their integration within healthcare settings, including issues related to cost, training, and workflow integration. Nonetheless, when combined with multidisciplinary approaches, these innovations facilitate communication and interaction among all healthcare professionals to maximize their potential.

## Figures and Tables

**Table 1:** Search strategy used in one of the databases––Medline by PubMed.

Search	Query ^1^	Records Retrieved
#1	“Reminder Systems” [Title/Abstract] OR “Reminder Systems” [MeSH Terms] OR “Electronics, Medical” [MeSH Terms] OR “Electronics, Medical [Title/Abstract] OR “Urinary Flow Measurement” [Title/Abstract] OR “Automatic Measurement of Urine [Title/Abstract] OR Urinometer [Title/Abstract] OR “Wireless Sensor [Title/Abstract] OR “Catheter Technology” [Title/Abstract] OR “Health Technology [Title/Abstract] OR “Digital Health” [MeSH Terms] OR “Digital Health” [Title/Abstract] OR “Remote Sensing Technology” [MeSH Terms] OR “Remote Sensing Technology” [title/Abstract] OR Technology [Title/Abstract]	237,879
#2	Infections [All Fields] OR Infections [MeSH Terms] OR “Urinary Tract Infections [All Fields] OR “Urinary Tract Infections [MeSH Terms] OR “Catheter-related Infections [All Fields] OR “Catheter-related Infections [MeSH Terms] OR “Infections Control” [MeSH Terms] OR “Infections Control” [All Fields]	1,116,844
#3	“Catheter, Indwelling” [Title/Abstract] OR “Catheter, Indwelling [MeSH Terms] OR “Urinary Catheter” [MeSH Terms] OR “Urinary Catheter” [Title/Abstract] OR “Urinary Catheterization” [Title/Abstract] OR “Urinary Catheterization [MeSH Terms] OR “Foley Catheter” [Title/Abstract] OR “Urethral Catheter” [Title/Abstract] OR “Bladder Catheterization” [Title/Abstract]	5672
#4	#1 AND #2 AND #3	39

^1^ Limited to language (English, Portuguese, Spanish).

**Table 2:** Joanna Briggs Institute method for assessing the articles’ level of evidence.

Article	Level of Evidence JBI [7]
Topal et al. [2]	Level 3.c––Cohort study with control group
Dong et al. [7]	Level 4.b––Individual diagnostic yield study
Fasugba et al. [3]	Level 1.c––Randomized Controlled Trial
Fukuda et al. [4]	Level 5.b––Expert consensus
Rea et al. [8]	Level 3.e––Observational study without a control group
Mitchell et al. [9]	Level 1.c––Randomized Controlled Trial
Xu et al. [10]	Level 4.b––Individual Diagnostic Yield Study

**Table 3:** Results of the articles included in the review.

Article (Autor/Year)	Method	Study Objective/Interventions	Sampling	Conclusions
Topal et al. (2019) [2]	Prospective Cohort Study	Investigate the incidence and infections associated with urinary catheters and the appropriate use of catheters in hospital settings.	Adult patients admitted in four medical units.	The study concluded that reducing the use of urinary catheters significantly decreased the incidence of CAUTIs. Nurses reported greater satisfaction with the technological method compared to the paper-based method.
Dong et al. (2022) [7]	Diagnostic Evaluation Study	Investigate the efficacy of rapid diagnostic approaches for UTIs using flow microimaging and artificial intelligence compared to traditional methods.	146 urine samples from adults with UTI suspicions.	The MUS-3600 device demonstrated a sensitivity of 72.9% and a specificity of 96.5% for nitrites and other bacteria counts, suggesting that this device has sufficient specificity to aid in clinical assessments and reduce unnecessary urine cultures.
Fukuda et al. (2020) [4]	Quantitative Study	Determine the concerns and perception of home-care nurses regarding the use of a mobile application for preventing and managing urinary catheter blockages.	26 home-care nurses from four home care units in Japan.	Interviews revealed several challenges, in particular limitations in the mobile application, data manipulation permissions, the need for more educational devices in training, multidisciplinary collaboration, data transmission security issues, and the lack of adaptability for caregivers and healthcare professionals.
Mitchell et al. (2019) [9]	Randomized Controlled Trial	Evaluate the effectiveness of the CATH TAG electronic reminder System in reducing urinary catheter duration.	1167 adult patients undergoing urinary catheterization.	The introduction of the CATH TAG system significantly reduced the duration of urinary catheterization and resulted in a 23% decrease in CAUTI incidence. The system was found to be easily applicable and accepted by healthcare professionals.
Nasrabadi et al. (2021) [1]	Integrative Literature Review	Address invasive and non-invasive methods for bladder volume monitoring, showing recent technologies like wearable devices.	Selection of studies, scientific articles, and publications related to bladder volume monitoring techniques, both invasive and non-invasive.	The study identified 15 wearable devices for bladder volume monitoring, though there were inconsistencies between theory and practice.
Xu et al. (2023) [10]	Retrospective Cohort Study	Assess whether biometric urine flow control improves bladder function in patients with long-term urinary catheterization.	30 patients in an ICU who required urinary catheterization for over 30 consecutive days.	The biometric urinary flow control technology demonstrated protective effects on bladder function after prolonged catheter use.
Rea et al. (2018) [8]	Quasi-Experimental Study	Assess the impact of software technology as a replacement for paper-based processes to improve nursing care quality in reducing CAUTI rates.	14 nurses provided information on CAUTI prevention according to evidence-based practice.	The study compared the manual paper-based record-keeping method with cloud-based technology. Nurses reported greater satisfaction with the technology due to its utility, clarity, and speed of data transmission.
Fasugba et al. (2018) [3]	Quantitative and Qualitative Study	Evaluates the effectiveness of the CATH TAG device in reducing urinary catheter use and its complications.	The sampling occurred in an Australian hospital over 24 weeks.	The study provided important data on the use of the CATH TAG device, showing its effectiveness in reducing catheter use and promoting patient safety and comfort.

**Table 4:** Impact of technologies on CAUTI prevention.

Authors	Method Addressed	CAUTI Prevention (Yes/No)	Commentary on the Results
Topal et al. (2019) [2]	Computerized feedback system; Nurse-directed protocol.	Yes	51% reduction in the number of catheterized patients; 42% reduction in catheter use during hospitalization; 81% reduction in CAUTIs per 1000 catheter days.
Dong et al. (2022) [7]	MUS-3600 system based on urine flow microimaging.	No	High specificity (96,5%) and reasonable sensitivity (72,9%) for UTI diagnosis; Reduction in unnecessary urine cultures.
Fukuda et al. (2020) [4]	Non-invasive method for preventing/reversing catheter blockages.	No	Prevention of obstructions in long-term catheters; Emphasizing the importance of ongoing nurse education.
Mitchell et al. (2019) [9]	CATH TAG electronic reminder system.	Yes	23% reduction in catheter use duration, leading to a decrease in CAUTI incidence.
Nasrabadi et al. (2021) [1]	Non-invasive technologies	No	Non-invasive alternatives for urine volume monitoring; Effective in less critical settings.
Fasugba et al. (2018) [3]	CATH TAG electronic reminder system.	Yes	Similar results to Mitchell et al., with a 23% reduction in catheter use, leading to a decrease in CAUTI incidence.
Xu et al. (2023) [10]	Biomimetic urinary flow device.	No	Technology that can simulate urethral sphincter function; Helps preserve bladder function in patients with long-term catheterizations.
Rea et al. (2018) [8]	Cloud-based software introduction.	No	Focus of evidence-based intervention and adherence to best practices; No specific data on CAUTIs.

**Table 5:** Synthesis.

Authors	Method Addressed	Advantages	Disadvantages
Topal et al. (2019) [2]	Multidisciplinary approach; Use of supporting technologies.	Reducing unnecessary use of urinary catheters; Decrease in CAUTIs; Reduction in hospital costs.	Resistance to electronic feedback system by some nurses; Need for continuous monitoring; Additional implementation costs.
Dong et al. (2022) [7]	MUS-3600 system based on urine flow microimaging.	High bacterial classification/counting ability; Diagnoses UTIs; High specificity; Reduces unnecessary urine cultures; Assists in antibiotic selection; Reduces hospital costs.	Despite high specificity, may not fully replace traditional urine analysis; No information on specificity for detecting more complex infections.
Fukuda et al. (2020) [4]	Mobile application.	Minimizes error risk; Improves efficiency during home care; Contributes to ongoing education and training	Increases workload; Not easily portable; No integration with other record system; Low security in information transmission.
Mitchell et al. (2019) [9]	CATH TAG electronic reminder system.	Reduction in catheterization duration; Easy to handle.	Overall reduction in catheterization was not statistically significant; Nurse perceptions varied across units, indicating potential barriers.
Nasrabadi et al. (2021) [1]	Non-invasive technologies	Less invasive; Wearable devices are easy to use; Low cost; Fewer side effects; Variety of devices available.	Risk of lower accuracy; Sensitivity to body movement/anatomy; Concerns about battery life, safety, and accuracy.
Xu et al. (2023) [10]	Biomimetic urinary flow device.	Maintains bladder function in patients with urinary catheterization; More controlled urine drainage; Contributes to improved quality of life.	Not described.
Rea et al. (2018) [8]	Cloud-based software introduction.	Provides real-time feedback; Easy data sharing; Higher nurse satisfaction; Improves compliance with evidence-based nursing practices; Reduces manual workload.	Additional costs; Requires adaptation to traditional practices; No demonstrated differences in preventing CAUTIs.
Fasugba et al. (2018) [3]	CATH TAG electronic reminder system.	Innovation approach; Reduction in catheter use duration; Easy to handle; Improvement in infection control.	Potential difficulties in implementation by nurses; Requires continuous evaluation; Shows only slight, not significant, reduction in catheter use.

## Data Availability

This study research is registered on the Platform Open Science Framework (OSF) https://doi.org/10.17605/OSF.IO/58APG under registration number ORCID 0009-0000-2383-5869, at 1 September 2024.

## Public Involvement Statement

No public involvement in any aspect of this research.

## Guidelines and Standards Statement

This manuscript was drafted based on the PRISMA (Preferred Reporting Items for Systematic Reviews and Meta-analyses) guideline, with adaptations for an integrative literature review [11].

## Use of Artificial Intelligence

In the preparation of the manuscript artificial intelligence-assisted (AI) tools were used. Specifically, AI was employed to translate sections of the manuscript from Portuguese to English, ensuring an accurate translation that preserved the original meaning. Additionally, we used the AI tools to review and correct grammatical issues, as well to improve clarity of the text, making it suitable for an English-speaking audience. These tools were instrumental in ensuring that the manuscript met the linguistic standards required for scientific publication in English, while maintain the integrity of the information presented.
